# Accuracy of hiatal hernia diagnosis in bariatric patients: Preoperative endoscopy *versus* intraoperative reference

**DOI:** 10.1002/jgh3.12388

**Published:** 2020-07-30

**Authors:** Daniel L Chan, Simon K‐H Wong, Hon Ting Lok, Jim Iliopoulos, Michael L Talbot, Annemarie Hennessy, Enders K‐W Ng

**Affiliations:** ^1^ Division of Upper Gastrointestinal and Metabolic Surgery, Department of Surgery, Faculty of Medicine The Chinese University of Hong Kong Hong Kong SAR China; ^2^ School of Medicine Western Sydney University Sydney New South Wales Australia; ^3^ Faculty of Medicine The University of New South Wales Sydney New South Wales Australia

**Keywords:** accuracy, bariatric surgery, endoscopy, hiatal hernia, obesity

## Abstract

**Background and Aim:**

Obesity is becoming increasingly prevalent in Asia. Bariatric surgery in the region is growing in popularity to reflect increasing demand. Hiatal hernia (HH) is common among the obese population. There is a lack of evidence comparing preoperative endoscopy against intraoperative findings as a standard of reference for HH diagnosis.

**Methods:**

This was a retrospective analysis of a bariatric procedure database from a single tertiary teaching hospital and high‐volume endoscopy center. Electronic medical records were reviewed. Endoscopy results were compared to intraoperative findings, and subgroup analysis of >2 cm hernias was performed. Sensitivity, specificity, predictive values, likelihood ratios, and global diagnostic test accuracy were calculated.

**Results:**

A total of 434 patients were eligible for this study, of which HH was detected in 37 patients (prevalence rate 8.55%). Mean age was 41.51 ± 11.07 years, and body mass index was 39.37 ± 5.67 kg/m^2^. Endoscopy sensitivity was 75.68% (95% confidence interval, 58.80–88.23%) and specificity 91.44% (88.24–94.00%). Positive likelihood ratio was 8.53 (6.11–12.79) and negative likelihood ratio 0.27 (0.15–0.47). Positive predictive value was 45.16% (36.27–54.38%) and negative predictive value 97.58% (95.80–98.62%). Accuracy of endoscopy for preoperative HH diagnosis was 90.09% (86.89–92.74%).

**Conclusion:**

Endoscopy can have a high diagnostic accuracy of preoperative HH diagnosis in obese Asian patients using intraoperative diagnosis as the reference standard.

## Graphical abstract

This is a retrospective analysis of a bariatric procedure database, with endoscopy results compared to intraoperative findings. A total of 434 patients were eligible, of which hiatal hernia was detected in 37 patients (prevalence rate 8.55%). Endoscopy sensitivity was 75.68% and specificity 91.44%. Endoscopy can have high diagnostic accuracy (90.09%) for preoperative hiatal hernia diagnosis in obese Asian patients.

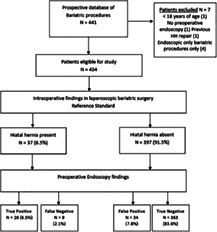

## Introduction

The prevalence of overweight and obesity is a global health issue, driven by urbanization, overnutrition, and sedentary lifestyle.^1^ Obesity‐related morbidity and mortality is well established, with concomitant comorbidities such as diabetes mellitus, cardiovascular disease, gallstone disease, osteoarthritis, chronic back pain, and several malignancies.^2^ Asia has been unable to avoid this global trend, with almost a third of the population classified as overweight or obese in some regions.^3^ There was a 400% increase in prevalence in China over the past two decades, compared to 20% in Australia over the same period.^4^

Bariatric surgery remains the only effective means of sustained weight loss and is associated with reduced overall mortality.^5^ Bariatric surgery in Asia continues to grow in popularity as a reflection of increasing demand. The most commonly performed procedures in the Asian region are laparoscopic sleeve gastrectomy (LSG), followed by laparoscopic Roux‐en‐Y gastric bypass (RYGB), one‐anastomosis gastric bypass (OAGB), and laparoscopic adjustable gastric banding (LAGB).^6^ These bariatric procedures involve dissection of the angle of His, which therefore provides an opportunity to visualize and repair hiatal hernias (HHs). This may be particularly important in LSG, where postoperative gastroesophageal reflux disease (GERD) is highly prevalent.^7^

HH is common in obese patients undergoing bariatric surgery. Preoperative HH diagnosis may guide meticulous intraoperative detection and repair of small HH, as well as facilitate preoperative decision‐making and improved patient consent. Intraoperative HH diagnosis by direct visualization has become an opportunistic reference standard in bariatric surgery. Previous comparative literature of preoperative assessment with endoscopy, barium swallow, and high‐resolution manometry (HRM) often lack intraoperative reference.^8^, ^9^ There is a paucity of literature assessing the accuracy of endoscopy in HH diagnosis and no previous studies on an Asian bariatric patient cohort.

## Methods

**Figure 1:**
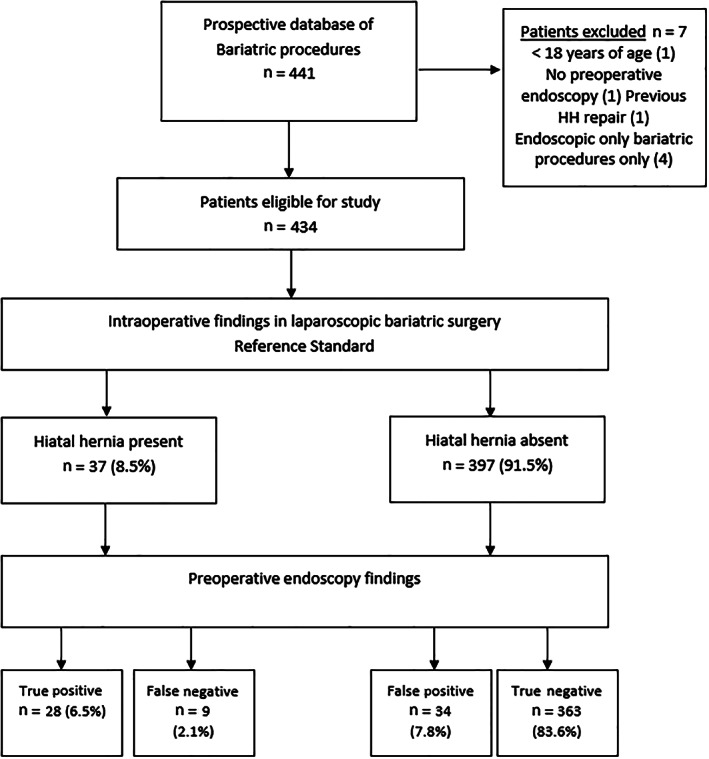
Study patient flow diagram. HH, hiatal hernia.

A retrospective analysis was performed on a prospective database of consecutive patients undergoing bariatric surgery from 2006 to 2019 in a single tertiary teaching hospital and World Endoscopy Organization Centre of Excellence. Further electronic medical record reviews of a regional electronic database were conducted to ensure comprehensiveness of data collection. Data points of interest included demographics, such as age, gender, and body mass index (BMI), preoperatively; pharmacotherapy for reflux; GERD; endoscopy results (HH presence and size); and intraoperative findings and type of bariatric surgery. Inclusion criteria were consecutive adult patients undergoing bariatric surgery with preoperative endoscopy. Exclusion criteria included patients aged <18 years of age (*n* = 1), no preoperative endoscopy result (*n* = 1), previous HH repair surgery (*n* = 1), and patients who underwent endoscopic bariatric procedures only (*n* = 4) (Fig. 1). Ethics approval was obtained from the hospital's institutional review board.

Endoscopic HH was diagnosed if the esophagogastric junction was ≥2 cm above the diaphragmatic pinch, using hash marks on an endoscope (spaced 5 cm apart). Endoscopic findings of HH were subcategorized as ≤2 cm or >2 cm. Intraoperative inspection of the hiatus for gross defect or dimpling anterior to the esophagus was conducted after routine exposure of the left crus. If present, the right crus was also exposed, and primary repair was performed. Routine dissection of both crus was not performed as this was not required for the procedures performed, and there are anecdotal concerns of the overestimation of HH and even predisposition to postoperative GERD.

Statistical analyses were performed using IBM SPSS Statistics for Windows, Version 25.0. (IBM Corp. Released 2017. Armonk, NY, USA: IBM Corp). Continuous variables were presented as mean ± SD (range) and categorical data as number (percentage). Comparative analysis was performed by *t*‐test, Chi‐squared, two‐way repeated measure ANOVA, and Mann–Whitney *U* test, where appropriate. Intraoperative findings were the reference (“gold standard”) of HH presence or absence. A *P*‐value of <0.05 was considered statistically significant. Sensitivity, specificity, predictive values, and accuracy were calculated and presented as percentages (95% confidence intervals [CI]). Positive and negative likelihood ratios and 95% CI were presented. Accuracy was calculated as (sensitivity x prevalence) + (specificity × [1 − prevalence]).^10^, ^11^

## Results

The total number of patients included in the study was 434 patients with 441 bariatric procedures in the study period (seven excluded). The mean patient age was 41.51 ± 11.07 (18–64) years. The majority of patients were female (248, 57.1%). The mean preoperative BMI was 39.37 ± 5.67 (26.46–59.25) kg/m^2^. Prevalence of preoperative GERD was 17.3% (*n* = 75), and preoperative pharmacotherapy for reflux was 8.5% (*n* = 37).

Intraoperative diagnosis confirmed that 37 patients had HH, resulting in a cohort prevalence of 8.55%. Operations performed were LSG (70.0%), RYGB/OAGB (29.3%), and LAGB (0.7%). Patients with HH were older (45.89 *vs* 41.10 years, *P* = 0.01), more likely to suffer from GERD (56.8 *vs* 13.6%, *P* < 0.01), and have pharmacotherapy for reflux (35.1 *vs* 6. 8%, *P* < 0.01) than those without. There were no significant differences in preoperative BMI (38.16 *vs* 39.48 kg/m^2^, *P* = 0.18) or gender (females 70.3 *vs* 55.9%, *P* = 0.09) (Table 1).

**Table 1:** Patient demographics

	Total cohort	Patient with HH	Patients without HH	*P*‐value
Patients	434 (100)	37 (8.5)	397 (91.5)	
Age (years)	41.51 ± 11.07	45.89 ± 10.57	41.10 ± 11.03	0.01
Gender (female)	248 (57.1)	26 (70.3)	222 (55.9)	0.09
BMI (kg/m^2^)	39.37 ± 5.67	38.16 ± 4.95	39.48 ± 5.73	0.18
Preoperative reflux pharmacotherapy	37 (8.5)	13 (35.1)	24 (6.0)	<0.01
Preoperative GERD	75 (17.3)	21 (56.8)	54 (13.6)	<0.01

BMI, body mass index; GERD, gastro‐esophageal reflux disease; HH, hiatal hernia.

Endoscopy detected HH in 62 (14.3%) patients. Of these patients, 28 (true positive) had HH confirmed on intraoperative findings, and 34 (false positive) did not have HH. Of the 372 patients with negative endoscopic diagnosis, nine (false negative) had intraoperative findings of HH. The remaining 363 (true negative) patients had both negative endoscopy and intraoperative findings (Table 2). The endoscopic sensitivity was 75.68% (95% CI 58.80–88.23%) and specificity 91.44% (95% CI 88.24–94.00%). The positive likelihood ratio was 8.53 (6.11–12.79) and the negative likelihood ratio 0.27 (0.15–0.47). The positive predictive value of endoscopy was 45.16% (36.27–54.38%) and negative predictive value 97.58% (95.80–98.62%). The accuracy of endoscopy for preoperative HH diagnosis was 90.09% (86.89–92.74%) in this study cohort.

**Table 2:** Intraoperative and endoscopy findings of hiatal hernia (HH)

	Intraoperative HH		
Endoscopy HH	Positive	Negative	
Positive	28	34	62
Negative	9	363	372
	37	397	434

Of the 62 patients with positive endoscopy findings, 5 did not have HH size (cm) recorded and were excluded from the subgroup analysis. Of the remaining 57 patients, 39 (68.4%) had small HH (≤ 2 cm), and 18 (31.6%) had at least a moderate‐sized HH (>2 cm). Applying the cut‐off of at least a moderate‐sized endoscopic HH, the sensitivity reduced to 32.43% (18.01–49.79%), but both specificity, 98.49% (96.74–99.44%), and accuracy, 92.85% (90.01–95.09%), improved (Table 3).

**Table 3:** Endoscopy accuracy for preoperative hiatal hernia (HH) diagnosis

	Endoscopy HH diagnosis (all)	Endoscopy HH (>2 cm) diagnosis
	Value (95% confidence interval)	Value (95% confidence interval)
Sensitivity (TP/TP + FN)	75.68% (58.80–88.23%)	32.43% (18.01–49.79%)
Specificity (TN/FP + TN)	91.44% (88.24–94.00%)	98.49% (96.74–99.44%)
Positive likelihood ratio (sensitivity/1 − specificity)	8.53 (6.11–12.79)	21.46 (8.55–53.86)
Negative likelihood ratio (1 − sensitivity/specificity)	0.27 (0.15–0.47)	0.69 (0.55–0.86)
Positive predictive value (TP/TP + FP)	45.16% (36.27–54.38%)	66.68% (44.36–83.40%)
Negative predictive value (TN/TN + FN)	97.58% (95.80–98.62%)	93.99% (92.59–95.13%)
Accuracy (sensitivity × prevalence) + (specificity × [1 − prevalence])	90.09% (86.89–92.74%)	92.85% (90.01–95.09%)

FN, false negative; FP, false positive; HH, hiatal hernia; TN, true negative; TP, true positive.

## Discussion

HHs occur when contents of the abdominal cavity protrude into the thorax through the elliptical esophageal hiatus. This displacement and negative thoracic pressure drives GERD and, less commonly, mass effect‐type respiratory and gastrointestinal symptoms, such as postprandial shortness of breath, chest fullness or pain, and even gastric volvulus.^12^ The wide range of reported HH prevalence (7.32–76.92%) is due to study design, selection bias, and geographical variability between studies.^13^, ^14^ Obesity increases intra‐abdominal pressure, intragastric pressure, and—in turn—increases gastroesophageal pressure gradient.^15^ There is a complex interplay between obesity, HH disease, and GERD symptoms.^16^, ^17^ To our knowledge, this study is the first to examine the diagnostic accuracy of preoperative endoscopy in an Asian bariatric cohort. In our cohort, increased age (*P* = 0.01), preoperative GERD (*P* < 0.01), and pharmacotherapy for reflux (*P* < 0.01) were significantly associated with intraoperative HH diagnosis.

LSG in particular can both exacerbate and potentiate de novo GERD development.^7^, ^15^ This is likely due to a combination of converting the stomach into a high‐pressure tubular structure with reduced compliance, potentially retaining gastric fundus and weakening or stretching of the phrenoesophageal ligament and separation of the surrounding crural muscle fibers, leading to HH.^18^ Therefore, accurate preoperative diagnosis is important to increase clinical suspicion of small HH and to avoid excessive intraoperative dissection in those patients without HH.

The role of preoperative endoscopy before bariatric surgery remains relatively controversial. Individual centers need to consider the prevalence of Barrett's esophagus with dysplasia, gastric malignancy, HH, and other management‐changing diagnoses to optimize resource utilization.^19^, ^20^ Early literature assessing HH diagnosis without an appropriate reference standard is not appropriate for assessment of diagnostic accuracy. When considering only studies that have used intraoperative diagnosis as the reference standard, preoperative HH diagnosis still has a wide range of sensitivity (36.72–96.23%), specificity (66.67–93.3%), and accuracy (45.38–89.16%).^13^, ^14^, ^21^, ^22^

Our center had a high sensitivity (75.68%), specificity (91.44%), and accuracy (90.09%) compared to the existing literature. This specificity (98.49%) and accuracy (92.85%) was further improved when considering only at least moderate‐sized HH (>2 cm) but with the compromise of reduced diagnostic sensitivity (32.43%). Small HH, as determined by endoscopy, are more likely to be false positives than those >2 cm; this is understandable given that the diaphragmatic pinch is a physiologically mobile reference point. The high diagnostic accuracy of endoscopy in our center is attributed to institutional experience and patient cohort. As a Centre of Excellence for digestive endoscopy, our center has a high volume of diagnostic and complex interventional endoscopy. In addition, disease prevalence affects the global measure of diagnostic accuracy. For the same sensitivity and specificity, the diagnostic accuracy of an investigation increases as the disease prevalence is reduced.^23^ The prevalence of HH in our cohort was 8.53%, which is lower than most other studies on this issue and is reflective of reduced GERD and HH prevalence among Asians.^24^, ^25^

The limitations of this study are the retrospective nature and potential risk of bias of the standard of reference. It is recognized that intraoperative findings may be subject to interobserver variability due to differences in patient positioning, CO_2_ insufflation, and an incomplete visualization despite standardization of technique. Despite the inherent limitations to this reference standard, direct intraoperative visualization of HH represents a practical reference for preoperative investigations. In addition, preoperative and postoperative GERD symptoms were not assessed within the design of this study. The variability in operative procedures performed and the low prevalence of HH in this cohort would have made findings difficult to interpret.

A recent meta‐analysis comparing barium swallow, HRM, and endoscopy for preoperative HH diagnosis suggested that manometry exhibited better diagnostic performance and that barium swallow and endoscopy were equivocal.^26^ However, it is noted that our endoscopy results fall within the range of the pooled sensitivity of 77% (95% CI 70–83%) and specificity of 92% (95% CI 85–96%) of HRM. This meta‐analysis also considered both bariatric and nonbariatric populations and only had a single study of 130 patients from the Asian region.^14^ When considering access and resource utilization of HRM, endoscopy can yield equivalent diagnostic accuracy in the context of a high‐volume endoscopy center. Although HRM provides useful information about hiatal anatomy and esophageal motility, endoscopy provides a breadth of diagnostic data and the opportunity for intervention. Endoscopy will likely remain the preferred preoperative screening test for patients before bariatric surgery in many international centers.

In conclusion, endoscopy can have high diagnostic accuracy for preoperative HH in obese Asian patients, using intraoperative diagnosis as the reference standard. Endoscopy can achieve an accuracy comparable to HRM in high‐volume endoscopy centers.

## References

[1] Chooi YC , Ding C , Magkos F . The epidemiology of obesity. Metabolism. 2019; 92: 6–10. 30253139 10.1016/j.metabol.2018.09.005

[2] Guh DP , Zhang W , Bansback N , Amarsi Z , Birmingham CL , Anis AH . The incidence of co‐morbidities related to obesity and overweight: a systematic review and meta‐analysis. BMC Public Health. 2009; 9: 88. 19320986 10.1186/1471-2458-9-88 PMC2667420

[3] Ko GT , Wu MM , Tang J , Wai HP , Chan CH , Chen R . Body mass index profile in Hong Kong Chinese adults. Ann. Acad. Med. Singapore. 2001; 30: 393–6. 11503547

[4] Collaboration APCS . The burden of overweight and obesity in the Asia‐Pacific region. Obes. Rev. 2007; 8: 191–6. 17444961 10.1111/j.1467-789X.2006.00292.x

[5] Sjostrom L , Narbro K , Sjostrom CD *et al* Effects of bariatric surgery on mortality in Swedish obese subjects. N. Engl. J. Med. 2007; 357: 741–52. 17715408 10.1056/NEJMoa066254

[6] Lomanto D , Lee WJ , Goel R *et al* Bariatric surgery in Asia in the last 5 years (2005–2009). Obes. Surg. 2012; 22: 502–6. 22033767 10.1007/s11695-011-0547-2

[7] Mahawar KK , Carr WR , Jennings N , Balupuri S , Small PK . Simultaneous sleeve gastrectomy and hiatus hernia repair: a systematic review. Obes. Surg. 2015; 25: 159–66. 25348434 10.1007/s11695-014-1470-0

[8] Hashmi S , Rao SS , Summers RW , Schulze K . Esophageal pressure topography, body position, and hiatal hernia. J. Clin. Gastroenterol. 2014; 48: 224–30. 24440930 10.1097/MCG.0000000000000057

[9] Fornari F , Gurski RR , Navarini D , Thiesen V , Mestriner LH , Madalosso CA . Clinical utility of endoscopy and barium swallow X‐ray in the diagnosis of sliding hiatal hernia in morbidly obese patients: a study before and after gastric bypass. Obes. Surg. 2010; 20: 702–8. 19756887 10.1007/s11695-009-9971-y

[10] Mercaldo ND , Lau KF , Zhou XH . Confidence intervals for predictive values with an emphasis to case‐control studies. Stat. Med. 2007; 26: 2170–83. 16927452 10.1002/sim.2677

[11] Griner PF , Mayewski RJ , Mushlin AI , Greenland P . Selection and interpretation of diagnostic tests and procedures. Principles and applications. Ann. Intern. Med. 1981; 94(4 Pt 2): 557–92. 6452080

[12] Siegal SR , Dolan JP , Hunter JG . Modern diagnosis and treatment of hiatal hernias. Langenbecks Arch. Surg. 2017; 402: 1145–51. 28828685 10.1007/s00423-017-1606-5

[13] Goitein D , Sakran N , Rayman S , Szold A , Goitein O , Raziel A . Barium swallow for hiatal hernia detection is unnecessary prior to primary sleeve gastrectomy. Surg. Obes. Relat. Dis. 2017; 13: 138–42. 27639982 10.1016/j.soard.2016.08.006

[14] Tuerdi B , Kuerbanjiang A , Tailaiti G , Zhang Y , He J , Abdurehim K . High value of high‐resolution manometry applied in diagnosing hiatal hernia compared with barium esophagogram and endoscopy: a single‐center retrospective study. Int. J. Clin. Exp. Med. 2018; 11: 3113–20.

[15] Melissas J , Braghetto I , Molina JC *et al* Gastroesophageal reflux disease and sleeve gastrectomy. Obes. Surg. 2015; 25: 2430–5. 26428250 10.1007/s11695-015-1906-1

[16] Pandolfino JE . The relationship between obesity and GERD: "big or overblown". Am. J. Gastroenterol. 2008; 103: 1355–7. 18510602 10.1111/j.1572-0241.2008.01916.x

[17] de Vries DR , van Herwaarden MA , Smout AJ , Samsom M . Gastroesophageal pressure gradients in gastroesophageal reflux disease: relations with hiatal hernia, body mass index, and esophageal acid exposure. Am. J. Gastroenterol. 2008; 103: 1349–54. 18510603 10.1111/j.1572-0241.2008.01909.x

[18] Stenard F , Iannelli A . Laparoscopic sleeve gastrectomy and gastroesophageal reflux. World J. Gastroenterol. 2015; 21: 10348–57. 26420961 10.3748/wjg.v21.i36.10348 PMC4579881

[19] Schneider R , Lazaridis I , Kraljevic M , Beglinger C , Wolnerhanssen B , Peterli R . The impact of preoperative investigations on the management of bariatric patients; results of a cohort of more than 1200 cases. Surg. Obes. Relat. Dis. 2018; 14: 693–9. 29519608 10.1016/j.soard.2018.01.009

[20] Salama A , Saafan T , El Ansari W , Karam M , Bashah M . Is routine preoperative esophagogastroduodenoscopy screening necessary prior to laparoscopic sleeve gastrectomy? Review of 1555 cases and comparison with current literature. Obes. Surg. 2018; 28: 52–60. 28685362 10.1007/s11695-017-2813-4

[21] Tolone S , Savarino E , Zaninotto G *et al* High‐resolution manometry is superior to endoscopy and radiology in assessing and grading sliding hiatal hernia: a comparison with surgical in vivo evaluation. United European Gastroenterol J. 2018; 6: 981–9. 10.1177/2050640618769160 PMC6137592 30228885

[22] Santonicola A , Angrisani L , Vitiello A *et al* Hiatal hernia diagnosis prospectively assessed in obese patients before bariatric surgery: accuracy of high‐resolution manometry taking intraoperative diagnosis as reference standard. Surg. Endosc. 2020; 34: 1150–6. 31139983 10.1007/s00464-019-06865-0

[23] Simundic AM . Measures of diagnostic accuracy: basic definitions. EJIFCC. 2009; 19: 203–11. 27683318 PMC4975285

[24] Wang A , Mattek NC , Holub JL , Lieberman DA , Eisen GM . Prevalence of complicated gastroesophageal reflux disease and Barrett's esophagus among racial groups in a multi‐center consortium. Dig. Dis. Sci. 2009; 54: 964–71. 19255852 10.1007/s10620-009-0742-3 PMC3856566

[25] Corley DA , Kubo A , Zhao W . Abdominal obesity, ethnicity and gastro‐oesophageal reflux symptoms. Gut. 2007; 56: 756–62. 17047097 10.1136/gut.2006.109413 PMC1954862

[26] Li L , Gao H , Zhang C *et al* Diagnostic value of X‐ray, endoscopy, and high‐resolution manometry for hiatal hernia: a systematic review and meta‐analysis. J. Gastroenterol. Hepatol. 2020; 35: 13–8. 31206788 10.1111/jgh.14758

